# Health Tract, No. 211

**Published:** 1865

**Authors:** 


HEALTH TRACT, No. 211.

### Eating Economically.

WHAT kind of food has the most nourishment and costs the least? is a question of great practical importance. The following tables may be studied with considerable interest by every family. They will show the mode of preparation, the amount of nutriment, and the time required for the digestion of the most common articles of food placed upon our tables. A dollar's worth of meat, at twenty-five cents a pound, goes as far as fifty cents' worth of butter, at half a dollar a pound. Three pounds of flour, at eight cents a pound, is said to contain as much nutriment as nine pounds of roast beef, which, at twenty-five cents, is $2 25; that is, twenty-five cents' worth of flour goes as far as nine times that much money spent for roast beef, as weighed at the butcher's stall. A pint of white beans, weighing one pound, and costing seven cents, contains as much nutriment as three pounds and a half of roast beef, costing eighty-seven and a half cents. Of all the articles that can be eaten, the cheapest are bread, butter, molasses, beans, and rice. A pound of corn meal (Indian) goes as far as a pound of flour; so that, fine family flour at sixteen dollars a barrel, in New-York City, in July, 1864, and corn-meal at four cents, the latter is just one half less expensive. If corn and wheat were ground, and the whole product, bran and all, were made into bread, fifteen per cent of nutriment would be saved, with much greater healthfulness. These are standard tables:

| *Quality of Food.* | *Mode of Preparation.* | *Amount of Nutriment.* | *Time of Digestion.* H. M. |
|---|---|---|---|
| Cucumbers, | Raw. | 2 per cent. | |
| Turnips, | Boiled. | 4 | 3.30. |
| Milk, | Fresh. | 7 | 2.15. |
| Cabbage, | Boiled. | 7 | 4.30. |
| Apples., | Raw. | 10 | 1.50. |
| Potatoes, | Boiled. | 13 | 2.30. |
| Fish, | Broiled. | 20 | 2.00. |
| Venison, | " | 22 | 1.30. |
| Pork, | Roasted. | 24 | 5.15. |
| Veal, | " | 25 | 4.00. |
| Beef, | " | 26 | 3.30. |
| Poultry, | " | 27 | 2.45. |
| Mutton, | " | 30 | 3.15. |
| Bread, wheat, | Baked. | 80 | 3.30. |
| Bread, corn, | " | 80 | 3.30. |
| Beans, | Boiled. | 87 | 2.30. |
| Rice, | | 88 | 1.00. |
| Butter and oils, | | 96 | 3.30. |
| Sugars and Syrups, | | 96 | 3.30. |

